# Italian consensus on pediatric myopia: findings from a three-round modified delphi study

**DOI:** 10.1007/s00417-026-07246-9

**Published:** 2026-04-30

**Authors:** Irene Schiavetti, E. Villani, M. Serafino, R. Caputo, L. Buzzonetti, A. Vagge, P. Nucci, Italian study group on pediatric myopia, Giacomo Bacci, Contanza Barresi, Luca Buzzonetti, Roberto Caputo, Carlo Catti, Alberto Cuccu, Marika Dello Strologo, Simone Donati, Liviana Fontanel, Antonio Frattolillo, Elena Gusson, Domenico Lepore, Rosa Longo, Adriano Magli, Maria Pia Manitto, Viola Marchiori, Luigi Massa, Cristina Menicacci, Paolo Nucci, Ciro Picardi, Silvia Pignatto, Stefano Pensiero, Sara Pini, Ugo Procoli, Luca Rombetto, Lelio Sabetti, Annabella Salerni, Matteo Scaramuzzi, Irene Schiavetti, Massimiliano Serafino, Monica Stoppani, Giuliano Stramare, Aldo Vagge, Paola Valente, Martino Vannucchi, Edoardo Villani, Andrea Zampieri, Elisabetta Zannin

**Affiliations:** 1 Department of Health Sciences, University of Genoa, Genoa, Italy; 2 IRCCS Azienda Ospedaliera Metropolitana, Genoa, Italy; 3 Department of Clinical Sciences and Community Health, University of Milan, Milan, Italy; 4 San Giuseppe Hospital, Eye Clinic, IRCCS Multimedica, Milan, Italy; 5 Department of Surgical Science, Division of Ophthalmology, IRCCS Istituto G. Gaslini, Genova, Italy; 6 Pediatric Ophthalmology, Meyer Children’s Hospital IRCCS, Florence, Italy; 7 Ophthalmology Unit, Bambino Gesù Children’s Hospital IRCCS, Rome, Italy; 8 DINOGMI, Eye Clinic, University of Genoa, Genoa, Italy; 9 Department of Biomedical, Surgical and Dental Sciences, University of Milan, Milan, Italy

**Keywords:** Pediatric myopia, Delphi consensus, Myopia control, Public health / screening

## Abstract

**Background:**

Pediatric myopia is a growing public health concern, influenced by genetic and environmental factors such as limited outdoor time and excessive near work. It entails long-term risks beyond refractive correction. Despite expanding evidence on pharmacologic and optical interventions, clinical practice remains variable and several operational issues persist.

**Objectives:**

To develop evidence-informed consensus statements on pediatric myopia management in Italy, covering epidemiology, risk factors, prevention, monitoring, policy, and economic aspects.

**Methods:**

Surveys were administered through REDCap between November 2024 and August 2025. Pediatric ophthalmologists rated domain-specific items using Likert scales and optional comments. After each round, anonymized summaries were shared. Items reaching consensus were removed; others were revised when appropriate.

**Results:**

All 37 ophthalmologists completed every round. Fifty-three statements reached consensus. Key recommendations included early screening at age three, specialized clinics, awareness campaigns, financial support, prioritizing outdoor activity, not recommending red-light therapy, endorsing simultaneous competitive defocus spectacle lenses, and monitoring via cycloplegic refraction and axial length every 6 months.

**Conclusions:**

This consensus offers a structured framework for Italian practice and policy, while highlighting priorities for future research.

**Supplementary Information:**

The online version contains supplementary material available at https://doi.org/10.1007/s00417-026-07246-9.

## Key messages

*****What is known***:**

- Pediatric myopia is an increasing public health concern associated with long-term ocular complications and significant socioeconomic burden.
- Pharmacological, optical, and lifestyle interventions can slow myopia progression, but clinical thresholds and implementation strategies remain heterogeneous across healthcare systems.

*****What is new***:**

- This is the first Italian national consensus on pediatric myopia developed through a structured three-round modified Delphi process involving 37 pediatric ophthalmologists.
- The consensus provides explicit clinical criteria for treatment initiation (≥0.50 D/year and/or ≥0.2 mm/year axial elongation), recommends 6-month monitoring, and identifies defocus-based spectacle lenses and low-dose atropine as preferred strategies within the Italian healthcare context.
- It integrates clinical recommendations with health policy priorities (early screening at age 3, school-based awareness programs, and economic support measures) and does not recommend red-light therapy due to insufficient long-term safety evidence.

**Supplementary Information:**

The online version contains supplementary material available at https://doi.org/10.1007/s00417-026-07246-9.

## Introduction

Myopia is one of the most pressing public health challenges in children and adolescents worldwide. Beyond its increasing prevalence, the condition brings significant long-term consequences: high myopia substantially elevates the lifetime risk of vision-threatening complications, including myopic macular degeneration, retinal detachment, glaucoma, and early cataract, with consequent impacts on quality of life and productivity [1, 2]. 

Furthermore, evidence indicates that these risks are degree-dependent: for example, a 1-diopter increase in myopia is associated with a 67% increase in the prevalence of myopic maculopathy [3]. Even, low and moderate myopia carry considerable risks. Moderate myopia (− 3.0 to − 6.0 D) has been associated with a higher likelihood of retinal detachment, glaucoma, and myopic maculopathy compared with emmetropic eyes, although the incidence is considerably lower than in high myopia [1]. Myopia thus represents “a continuum” of risk rather than a harmless refractive state.

Its cumulative impact on the population makes the increasing prevalence of myopia a major public health problem worldwide. Although initially viewed as a predominantly East Asian problem, recent evidence shows that childhood myopia is rising also across Europe with significant health-system and social implications [4].

This growing burden reflects the interaction between genetic predisposition and some modifiable environmental factors: urban living, intense educational demands, reduced outdoor time, and increased near work or screen use are consistently associated with myopia development, although their relative contributions differ by setting [5]. Recent pooled analyses indicate that higher daily screen exposure is associated with greater odds of myopia, with particularly strong associations for computer use[6]; each additional hour of screen time per day significantly increases the likelihood of myopia onset [7]. In contrast, interventions (often school-based) promoting increased time spent outdoors have been shown to reduce the incidence of myopia and slow myopic progression in refractive error [8–11]. In Italy, studies conducted after the COVID-19 lockdowns reported a significant myopic shift among primary-school children, concurring with increased near work and reduced outdoor time, and highlighting the importance of modifiable behaviors in the Italian context [12, 13].

During the past two decades, the management paradigm for pediatric myopia has shifted from passive refractive correction to active interventions aimed at slowing axial elongation.

A comprehensive overview of currently available interventions and their relative efficacy have recently been provided in the updated Cochrane living systematic review and network meta-analysis, as well as in the 2025 International Myopia Institute (IMI) report on interventions for controlling myopia onset and progression [14, 15]. These analyses support the effectiveness of pharmacological, optical, and environmental interventions in reducing myopia progression, although differences in study design, follow-up duration, and outcome reporting are still evident.

Pharmacologic approaches, such as low-dose atropine, demonstrate dose-dependent efficacy across multiple randomized controlled trials and longer-term studies, although optimal concentration and personalized response are still debated [16–18]. Orthokeratology has demonstrated significant reductions in axial elongation in randomized clinical trials, including the ROMIO study and subsequent controlled European trials [19, 20]. Spectacle and contact-lens innovations provide non-pharmacologic options: dual-focus soft contact lenses have shown multi-year efficacy in a randomized controlled clinical trial [21]. Spectacle lens provide non-pharmacologic options for myopia control. Defocus Incorporated Multiple Segments (DIMS) lenses have demonstrated significant reductions in refractive progression and axial elongation in randomized clinical trials and real-world studies [22–25].

Highly Aspherical Lenslet (HAL) lenses have demonstrated sustained efficacy in multi-year randomized clinical trials and in European pediatric cohorts [22, 26].

Comparative European data indicate that defocus-based spectacle lenses, low-dose atropine (0.1 mg/mL − 0.01%), and combination approaches can significantly reduce myopia progression and axial elongation compared with single-vision correction, with some studies reporting enhanced refractive outcomes with combination therapy at 12 months [25].

In addition, other optical designs, including the Cooperative Aberration Control for Myopia (CARE) lens and Defocus Optimized Technology (DOT) lenses, have demonstrated promising short- to medium-term efficacy in randomized or controlled clinical studies [27, 28].

Emerging technologies such as repeated low-level red-light therapy have shown short-term efficacy signals but require longer-term follow-up and careful safety monitoring before routine use [29, 30].

In this scenario, however, clinical practice remains heterogeneous despite growing evidence, and some questions persist: whom to treat and when to start? How to personalize the treatment according to risk phenotype? How to define and monitor response? When to discontinue, if necessary?

The IMI position papers have proposed common terminology and thresholds for clinical and epidemiologic studies [31]. Recent modified-Delphi and expert consensus initiatives conducted in Europe and in the UK and Ireland show that, where evidence is limited or inconsistent, agreement on recommendations tends to be weaker or still variable, highlighting the need for structured, multidisciplinary processes to inform clinical care. The European consensus also proposed a preliminary clinical algorithm to guide decision-making in pediatric myopia management [32, 33].

An overview of Italian society guidance promotes pediatric vision screening, recommending proactive detection routine check-ups and in preschool and school settings. However, surveillance remains fragmented and standardized national protocols for myopia are not yet well harmonized [34–36]. Moreover, Italian and European real-world cohorts have begun to document post-pandemic refractive shifts and to evaluate the effectiveness of myopia-control interventions in local practice [12, 13, 25] In addition, regulatory developments in Europe, such as the recent EMA authorization of atropine 0.01% ophthalmic solution for slowing myopia progression in children, will influence discussions on availability and reimbursement within the Italian health-care system [37].

In summary, the rising prevalence of myopia, together with modifiable risk factors, diversifying therapeutic possibilities and variable guidance, highlight the need for context-sensitive consensus to guide clinical and policy decisions.

While international reports provide general recommendations, their implementation must be adapted to national healthcare systems, regulatory environments, and available resources. A structured national consensus can help translate evidence into clinical practice within the local context.

Therefore, we conducted a three-round modified Delphi study involving Italian pediatric ophthalmologists, with the aim of developing consensus statements covering epidemiology, aetiology and risk factors, prevention, monitoring and control, policy, and economics in pediatric myopia. Our aim is to synthesize the expert opinion into clear, context-specific statements that can guide clinical practice while also identifying priorities for future research and policy action.

## Materials and methods

### Study design

We conducted a three-round modified Delphi study between November 2024 and August 2025 to establish consensus among ophthalmology experts on the epidemiology, aetiology and risk factors, prevention, management, policy, and economic aspects of paediatric myopia. The Delphi technique is a structured consensus-building process that uses iterative rounds of questionnaires, controlled feedback, and anonymity to minimize dominance bias and to promote convergence of expert opinions [38–40]. This methodology has been widely applied in clinical research to develop diagnostic criteria, treatment recommendations, and also public health strategies [41–46].

Clinical trial number: not applicable.

## Expert panel

A national panel of 37 clinical experts was selected according to predefined criteria, including authorship of peer-reviewed publications on myopia or related fields, active involvement in clinical care, epidemiological research or public health initiatives, membership in professional or scientific societies. Although all panelists were pediatric ophthalmologists, their expertise covered a broad variety of areas reflecting the multidisciplinary topics discussed in the six thematic round tables. In addition, an independent methodologist supported the process but did not participate in rating or consensus decisions.

## Preparatory consensus meeting

The Delphi process was preceded by a face-to-face consensus-building meeting, during which experts (who later participated in the Delphi process) were assigned to six thematic roundtables (epidemiology, health economics, health policy, aetiology and risk factors, myopia control, and prevention). Each roundtable was coordinated by a facilitator with recognized expertise in the respective area. Initial inputs from the discussions were summarized by the facilitators and consolidated by the methodologist, who drafted the operational definitions, the analysis plan, and the Round-1 questionnaire.

## Delphi procedure

The Delphi process was conducted in three consecutive rounds and implemented through the REDCap platform (Research Electronic Data CAPture), a secure web-based application designed to support data capture for research studies [47]. Personalized invitations were used to access the survey.

At the end of each round, ophthalmology experts received individual feedback from the methodologist, including their own responses and an anonymized summary of the panel’s overall results presented in tables and graphs. This iterative feedback enabled panellists to re-examine their positions in light of the group’s opinion while maintaining full anonymity. The flow of the rounds is illustrated below:

- Round 1 (exploratory): the initial questionnaire included items across six thematic domains and an additional section addressing general-interest issues. Different response formats were used, including binary, single-choice, multiple-choice, Likert-scale, continuous, and open-ended questions. The open-ended section was specifically designed to capture further concepts suggested by the experts, which were subsequently transformed into structured items for later stages.
- Round 2 (refinement): based on responses and comments from Round 1, some items were rephrased for clarity or merged when overlapping in content, while those already reaching consensus or clear rejection were excluded. All items in this round were presented in closed format.
- Round 3 (confirmation): this round focused on items that had not yet reached consensus. These statements, again presented in closed format, were re-presented to experts in order to achieve a final decision, either acceptance or exclusion.

At the end of the Delphi process, the statements that had reached consensus were reorganized within the six initial thematic domains and circulated once more to experts for a final reading. This last step was intended exclusively to confirm the clarity of the phrasing, with no changes to content already approved in previous rounds.

## Consensus criteria

Consensus thresholds were defined a priori according to published recommendations for Delphi studies in health research [39, 40]. Different thresholds were applied depending on the response format, reflecting the varying degrees of agreement typically achievable across item types. Specifically:

- Binary and single-choice items: consensus reached if ≥ 80% of respondents selected the same option.
- Likert-scale items: consensus reached if ≥ 70% convergence on one category, due to the greater dispersion usually observed in ordinal scales.
- Multiple-choice items: “Accepted” if selected by ≥ 80%, “Rejected” if < 50%, “Reconsidered” otherwise. In round 3, only “Accepted” or “Rejected” were retained.
- Ranking tasks: inter-rater agreement was assessed using Kendall’s W coefficient of concordance, with values ≥ 0.70 considered indicative of consensus.
- Open-ended responses were used only in the Round 1 and analyzed using text-mining techniques (tokenization, stop-word removal, frequency and dispersion analysis), and themes mentioned by at least 10% of experts were retained to inform the subsequent Delphi round.
- Continuous variables: consensus reached when ≥ 70% of responses fell within a predefined range around the median.

### Data analysis

All analyses were performed in R (version 4.1.3). Frequencies and percentages were used for categorical variables, medians and interquartile ranges for continuous items, and Kendall’s W was calculated to assess inter-rater agreement for ranking tasks. Results were summarized in tables and visualized through bar plots, histograms, and word clouds.

## Ethical considerations

As no patients were involved, ethical committee approval was not required. Participation of the panellists was voluntary, and completion of each questionnaire was considered implied consent.

## Results

### Panel characteristics

All 37 invited experts participated in the three Delphi rounds, resulting in a 100% response rate with no dropouts. Demographic and professional characteristics of the panel are summarized in Table 1.

**Table 1:** Ophthalmologist experts’ characteristics

Age, years		49.5 ± 11.85 50.0 (31.0–73.0)
Gender		
	Female	15 (40.5%)
	Male	22 (59.5%)
Geographic area of primary work		
	Northern Italy	23 (62.2%)
	Central Italy	9 (24.3%)
	Southern Italy	3 (8.1%)
	Italian Islands	2 (5.4%)
Formal subspecialty in ophthalmology		23 (62.2%)
Academic position		13 (35.1%)
Work setting (one or more)		
	Public hospital	14 (37.8%)
	University hospital	22 (59.5%)
	Private clinic	8 (21.6%)
	Research institute	4 (10.8%)
Children/adolescents personally followed with active myopia management strategies (last 5 years)		
	11–50	4 (10.8%)
	51–200	12 (32.4%)
	> 200	21 (56.8%)
Participation in myopia-related research projects		19 (51.4%)
Authored/co-authored of peer-reviewed publications on myopia		11 (29.7%)
Submitted grant application for myopia-related project (PI or co-applicant)		2 (5.4%)
Contribution to protocol, clinical guideline, or decision algorithm for myopia management		18 (48.6%)
Professional relationships/collaborations with companies in myopia treatments/diagnostics		8 (21.6%)

The mean age was 49.5 years (SD 11.9, range 31–73), and 15 experts (40.5%) were female. Most panelists worked in Northern Italy (62.2%), 23 (62.2%) reported a formal subspecialty in ophthalmology, and 13 (35.1%) held an academic position. The majority were employed in university hospitals (59.5%) or public hospitals (37.8%). Regarding clinical experience, all provided direct patient care for pediatric myopia, and 21 (56.8%) had followed more than 200 children or adolescents in the last five years with active myopia management strategies.

In terms of research activity, 19 (51.4%) had participated in myopia-related projects, 11 (29.7%) participated as authors in peer-reviewed publications, and 2 (5.4%) had submitted a grant application. Eighteen of them (48.6%) reported contributions to clinical guidelines or decision algorithms, and 8 (21.6%) declared professional collaborations with pharma companies involved in myopia.

### Delphi process

The initial questionnaire generated after the preparatory meeting underwent progressive refinement across three Delphi rounds. The number of items decreased after each iteration, reflecting acceptance, exclusion, or merging of overlapping concepts. At the end of the process, a total of 53 consensus statements were retained. Because of the mixed structure of the questionnaire (i.e., multiple-choice, Likert-scale, and open-ended), the number of statements achieving consensus at each round could not be precisely quantified. Indeed, multiple-choice items could reach consensus for some response options but not for others, while open-ended questions from Round 1 were designed to generate new items rather than to reach consensus at that stage. In several cases, final statements resulted from the synthesis of related items showing partial agreement on different aspects (for example, ≥ 80% on one factor and 75% on another), subsequently merged into a single statement. Items already accepted or excluded were not re-presented in subsequent rounds, explaining the smaller number of items in Round 3.

Therefore, neither the proportion of agreement nor the specific round of consensus can be precisely attributed to each final statement, as many resulted from iterative combination and reformulation across stages.

In the final validation step, conducted after Round 3, all 53 statements were unanimously approved (100%) by the panelists in their final wording.

**Fig. 1:**
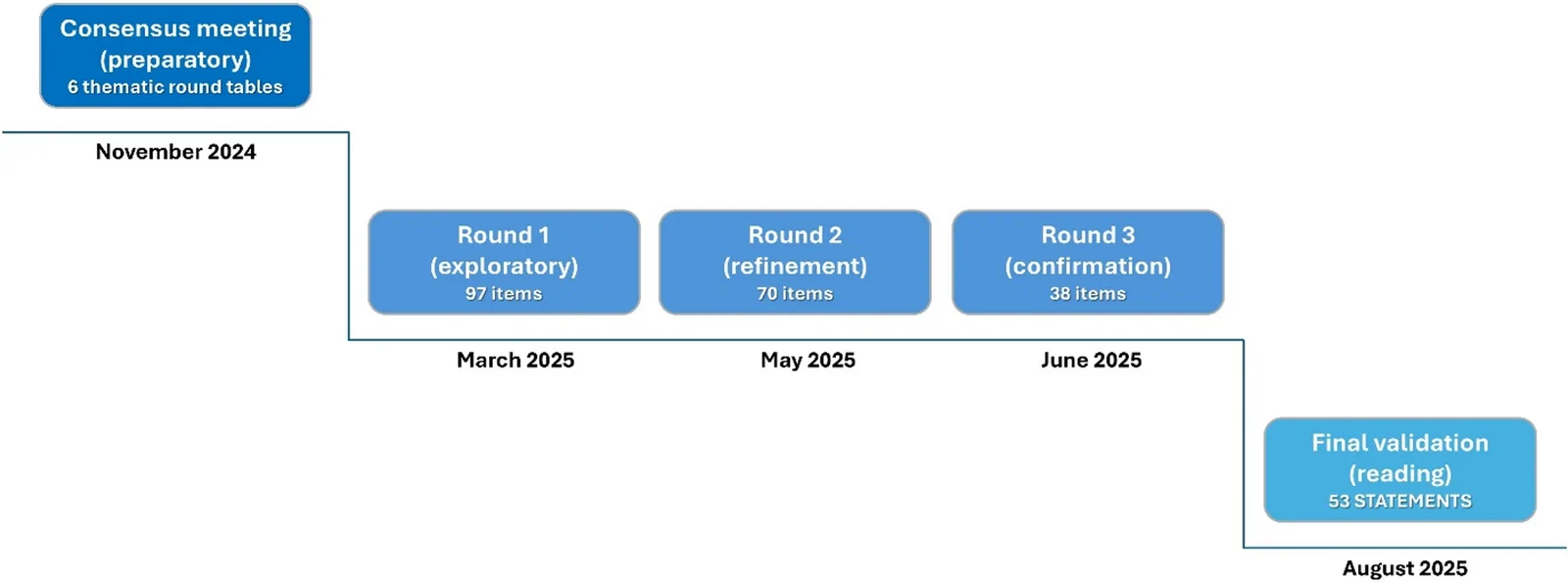
Flow of the Delphi process

The workflow and item count across rounds are illustrated in Fig. 1.

### Final consensus statements

**Fig. 2:**
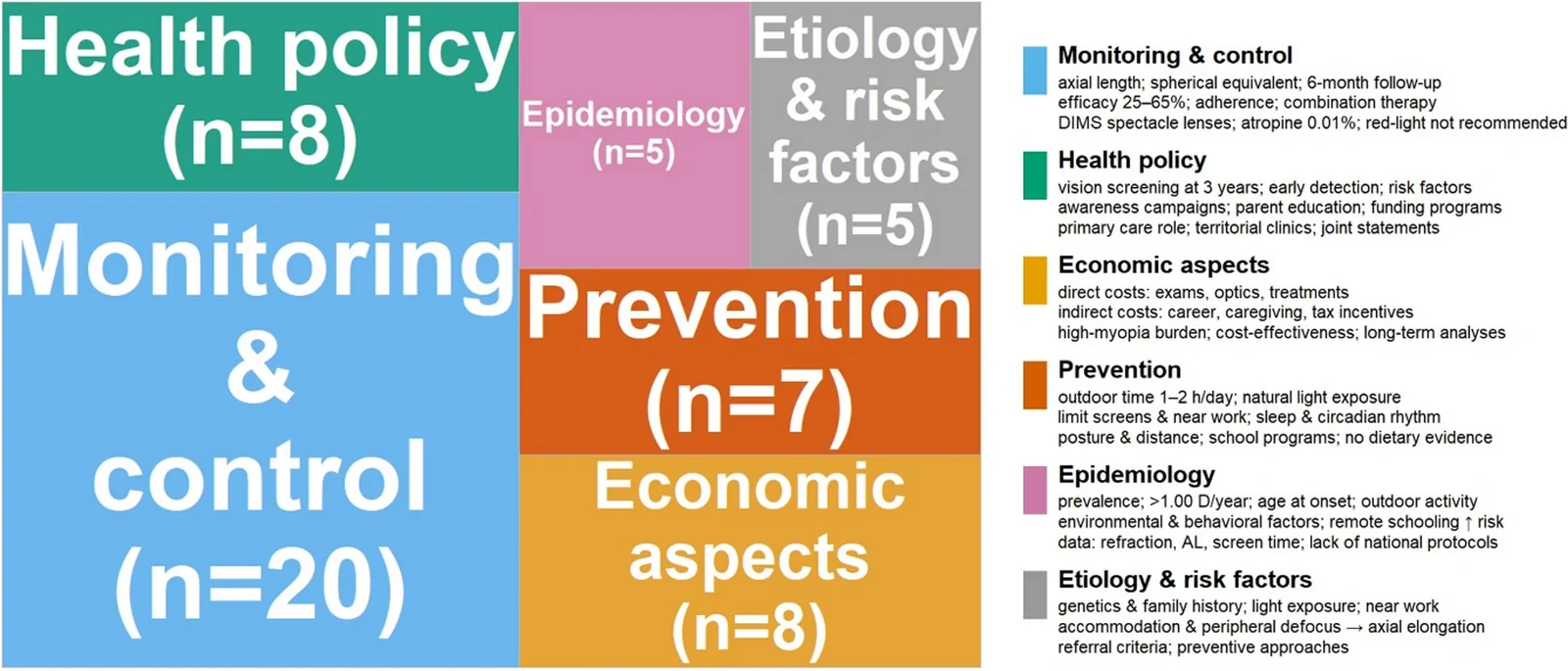
Six thematic areas

Table 2 reports the final statements retained after the Delphi process, organized into six thematic areas (Fig. 2).

**Table 2:** Final statements

HEALTH POLICY
1. Introducing vision screening at the age of three is useful for the early diagnosis and treatment of visual deficits such as myopia, amblyopia, and strabismus, as well as for the early identification of risk factors such as family history or improper use of digital devices.
2. Access to eye care for children with myopia can be improved through the implementation of specialized territorial clinics and the creation of multidisciplinary pediatric networks.
3. National awareness campaigns are useful for reducing myopia progression and should aim to inform families, schools, and pediatricians about the risks of myopia and prevention strategies, while also promoting healthy lifestyles such as increasing time spent outdoors, limiting near work, and using digital devices appropriately. Such campaigns should also include information on risk factors and possible complications associated with myopia, in order to improve public awareness and engagement.
4. In addition to tax deductions for spectacles and contact lenses, public funding should be provided to support programs aimed at preventing and managing myopia.
5. Ophthalmologists and primary care pediatricians should be recognized as key promoters of school-based campaigns to raise awareness about myopia and encourage healthy visual habits in children.
6. The presence of myopia in children and adolescents can significantly affect self-esteem, social relationships, and daily or school activities.
7. Primary care pediatricians should have a central role in educating parents about children’s visual health.
8. A joint statement from scientific societies is a useful tool to increase pediatricians’ awareness of the importance of early vision screening.
ECONOMIC ASPECTS
1. Regular eye examinations, corrective devices (such as spectacles and contact lenses), and pharmacological or optical treatments (such as low-dose atropine, orthokeratology, and simultaneous competitive defocus spectacle lenses) are among the main direct costs associated with myopia.
2. The costs of regular eye examinations and treatments aimed at reducing myopia progression are a justified preventive investment.
3. The economic burden of pathological myopia has a significant impact both on quality of life and on healthcare costs, and should be considered a priority in prevention and management strategies.
4. Indirect costs of myopia, such as career impact or informal caregiving, are not adequately addressed in current economic evaluations.
5. Complications of high myopia, such as myopic macular degeneration, retinal detachment, myopic choroidal neovascularization (CNV), and glaucomatous damage, have the greatest impact on patients’ quality of life and generate the highest direct and indirect costs for the healthcare system.
5. Complications of high myopia, such as myopic macular degeneration, retinal detachment, myopic choroidal neovascularization (CNV), and glaucomatous damage, have the greatest impact on patients’ quality of life and generate the highest direct and indirect costs for the healthcare system.
6. Simultaneous competitive defocus spectacle lenses are among the most cost-effective strategies for managing myopia in children and represent one of the most promising technological innovations to reduce its economic impact. Low-dose atropine and lifestyle modifications, such as increasing time outdoors and reducing near work, are also cost-effective interventions.
7. Long-term economic evaluations, which account for the prevention of complications and future savings, are essential to estimate the real cost-effectiveness of emerging treatments for myopia.
8. Tax deductions for healthcare expenses (including eye examinations, optical corrections, and pharmacological treatments) are the most effective economic measure to improve access to both conventional and innovative treatments for myopia.
PREVENTION OF MYOPIA
1. Recommending 1 to 2 h per day of outdoor activity is an appropriate preventive measure to reduce the onset of myopia in children.
2. Exposure to natural light contributes to myopia prevention by stimulating the release of retinal dopamine, which plays a protective role against axial elongation.
3. Limiting recreational screen time, promoting correct visual behaviors (such as appropriate posture, adequate reading distance, and avoiding improper use of digital devices), and increasing time spent outdoors are key strategies to reduce risks associated with near work in children. These measures should also be supported in schools through educational programs, modifications of spaces and activities, and the reduction of non-essential screen use during school hours.
4. In the school setting, raising awareness among students, teachers, and families about the importance of healthy visual habits is essential for effective myopia prevention.
5. According to the IMI definition, a refractive error between + 0.75 D and − 0.50 D, or below age-related norms, can represent pre-myopia. However, initiating treatment is ethically appropriate only in the presence of well-defined individual risk factors, such as altered biometric parameters or strong family history.
6. Respecting circadian rhythms and ensuring adequate sleep are useful factors in the prevention of myopia.
7. At present, there is no solid scientific evidence supporting a role for nutrition or dietary supplements in myopia prevention.
MONITORING AND CONTROL OF MYOPIA PROGRESSION
1. The decision to initiate treatment for myopia progression should be based on a combination of factors, including a significant progression rate, patient age, family history of myopia, type of myopia (e.g., high or pathological), presence of additional risk factors, patient and family compliance, and the existence of specific guidelines or clinical recommendations.
2. The main clinical parameters to be monitored for evaluating treatment effectiveness are cycloplegic spherical equivalent refraction and axial length.
3. An axial length progression rate higher than expected despite treatment is a strong indicator of therapeutic failure and represents the most relevant criterion for identifying a non-responder to myopia control therapy.
4. A treatment is ineffective when the annual progression in spherical equivalent refraction exceeds target values and/or when axial length increases beyond expected thresholds.
5. Patients under treatment should undergo follow-up visits approximately every 6 months.
6. According to the literature, acceptable efficacy ranges for treatments are a minimum 25–35% reduction and a maximum 55–65% reduction in myopia progression compared with untreated controls.
7. One of the main factors reducing the effectiveness of treatments (optical or pharmacological) is poor adherence.
8. The choice of ophthalmic lenses for myopia control should be based primarily on published scientific evidence and recommendations from scientific societies.
9. Simultaneous competitive defocus spectacle lenses represent the most appropriate optical technology for myopia control in children, as they are currently the only type of lens supported by solid scientific evidence.
10. Initiation of treatment with simultaneous competitive defocus spectacle lenses is appropriate in children aged 6–8 years with myopia progression ≥ 0.50 D/year (assessed over 6–12 months) and/or axial length increase ≥ 0.2 mm/year.
11. There is no universally recognized threshold in spherical equivalent refraction or axial length for discontinuing treatment with simultaneous competitive defocus spectacle lenses.
12. An absolute reduction in the annual myopia progression rate of at least 0.50 D in spherical equivalent refraction and/or at least 0.2 mm in axial length represents the threshold for defining a lens as effective for myopia control.
13. It is acceptable to suspend the use of myopia control spectacles during sports activities for up to two hours, up to three times per week.
14. Atropine 0.01% is the most commonly used initial concentration for myopia control, although there is no standardized strategy for dose adjustment over time and clinical practice is heterogeneous. The choice of concentration is guided mainly by the available scientific evidence, and treatment is generally continued until myopia stabilizes.
15. There is no universally accepted maximum age for initiating low-dose atropine treatment; initiation can be considered as early as 4–5 years of age, but in practice it is more often started between 6–8 years in the presence of documented axial length progression ≥ 0.2 mm/year. However, the decision should be based on a personalized clinical assessment, including progression rate, age, and family history, rather than on fixed refractive error thresholds. Exclusive use of atropine in cases of progression ≥ 0.5 D/year remains controversial.
16. Ethnicity and family history may influence decisions regarding the age thresholds for initiating or discontinuing atropine treatment.
17. After discontinuation of treatment with simultaneous competitive defocus spectacle lenses or atropine, it is advisable to schedule follow-up visits every six months to monitor possible myopia progression.
18. In addition to progression parameters, patient age and family history of myopia are important clinical factors guiding the choice between atropine and simultaneous competitive defocus spectacle lenses.
19. Combination therapy (e.g., atropine plus simultaneous competitive defocus spectacle lenses) is appropriate in cases of inadequate response to monotherapy and should be personalized according to clinical characteristics, such as rapid progression or high baseline myopia.
20. Red-light therapy and other emerging treatments are not currently recommended in clinical practice due to insufficient evidence.
EPIDEMIOLOGY
1. The proportion of children diagnosed with myopia, the percentage showing progression greater than 1 diopter per year, and the mean age at diagnosis are all relevant epidemiological indicators for monitoring the prevalence and course of pediatric myopia.
2. The course of myopia in childhood is strongly influenced by environmental and behavioral factors associated with urbanization, as well as school-related habits such as reduced time outdoors and prolonged near work.
3. Changes associated with remote schooling, as occurred during the COVID-19 pandemic, significantly increased the risk of myopia onset and progression. In longitudinal studies, it is essential to collect metrics such as cycloplegic refraction, axial length, time spent outdoors, and frequency and duration of digital device use in school settings. To develop reliable predictive models, data on screen exposure, age at diagnosis, annual progression, environmental factors, and family history or genetic predisposition should also be included.
4. Children with a positive family history of myopia and those with early or intensive exposure to digital devices are the most vulnerable subgroups for developing myopia.
5. Epidemiological surveillance is a valuable strategy to support the prevention of pathological myopia. School-based screening programs are the most effective setting for early identification and standardized data collection; however, the absence of national standardized protocols remains a major barrier to systematic data collection on myopia in Italy.
ETIOLOGY AND RISK FACTORS
1. The onset of myopia is influenced by genetic factors, exposure to natural light, time spent outdoors, and near work.
2. Specific genetic polymorphisms located in certain genomic loci are associated with the onset of myopia.
3. A detailed family and/or systemic clinical history is a useful clinical assessment to determine whether a child with myopia should be referred for specialist evaluation.
4. Ocular accommodation may contribute to the risk of developing myopia. Another mechanism appears to involve peripheral hyperopic defocus, which may stimulate axial elongation. When accommodation plays a role, preventive strategies aimed at its management are considered relevant.
5. Spending time outdoors and being exposed to natural light are recognized strategies to prevent the onset of myopia. Outdoor activity is particularly effective in preventing the onset of myopia rather than slowing its progression.

**Fig. 3:**
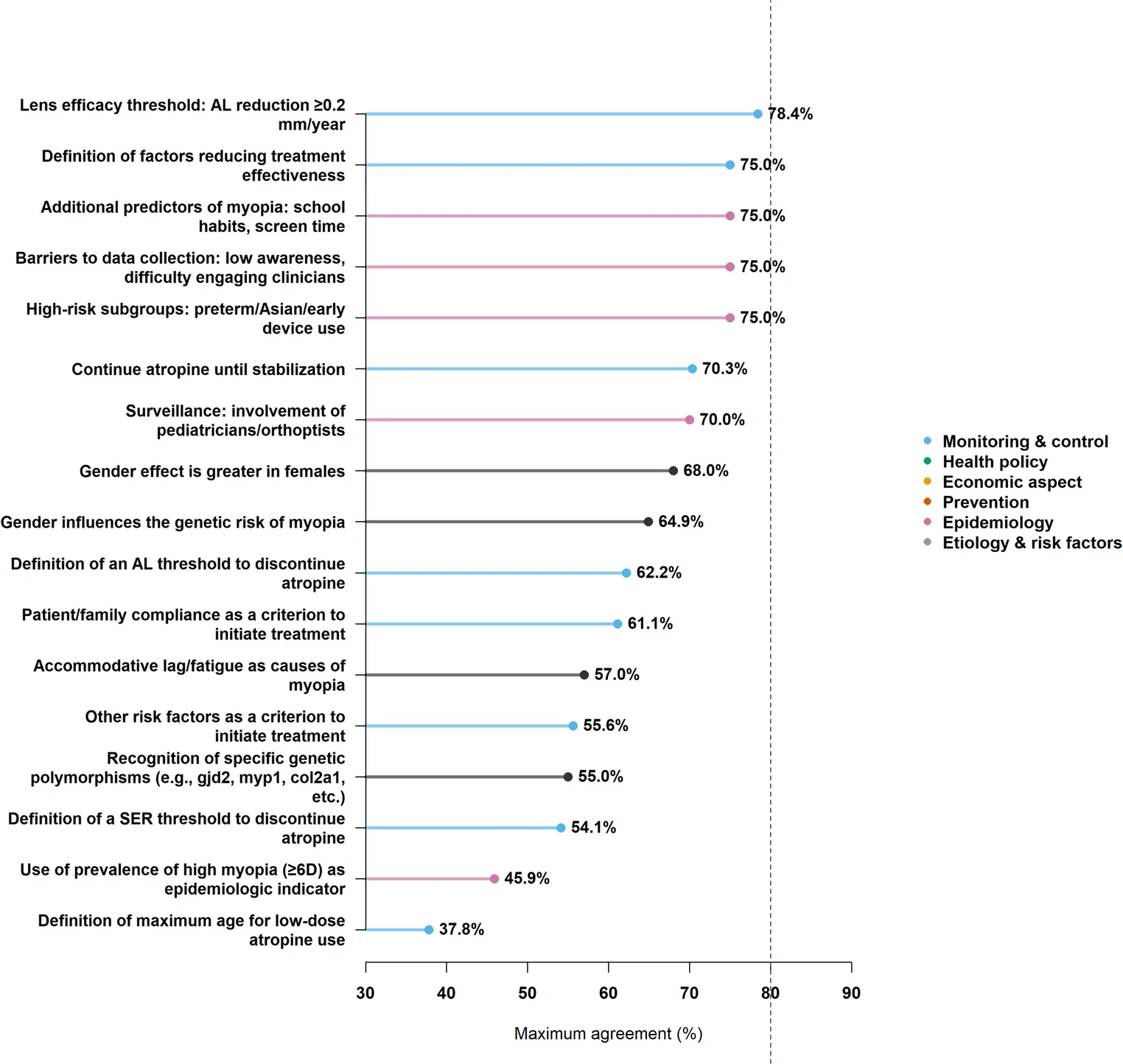
Statements without consensus

Within health policy (8 statements), the panelists emphasized the importance of early vision screening, improved access to care through territorial clinics and multidisciplinary networks, and the implementation of national educational campaigns aimed at families, schools, and pediatricians. Financial support, including public funding and tax deductions, was also considered important, together with the role of pediatricians and ophthalmologists in promoting healthy visual habits, the recognition of the psychosocial impact of myopia on children and adolescents, and the contribution of shared statements from scientific societies to raise awareness among pediatricians (Fig 3).

Economic aspects (8 statements) focused on both direct and indirect costs of myopia, highlighting the significant burden of complications such as myopic macular degeneration, retinal detachment, and glaucoma. The panel underlined the cost-effectiveness of optical and pharmacological treatments, including simultaneous competitive defocus spectacle lenses and low-dose atropine, as well as preventive lifestyle measures such as outdoor activity. Long-term economic evaluations were considered essential, and tax deductions were the most effective measure to improve access to both conventional and innovative care.

In the area of prevention (7 statements), outdoor activity and natural light exposure were consistently recognized as protective factors, while limiting recreational screen time and promoting correct visual behaviors should be recommended both in family and school contexts. The experts also agreed on the clinical relevance of the IMI definition of pre-myopia, while emphasizing that treatment initiation should be restricted to children with clearly defined additional risk factors. They also recognized the relevance of circadian rhythms and adequate sleep, and highlighted the absence of evidence supporting a role for nutrition or supplements.

Monitoring and control of myopia progression (20 statements) themes covered criteria for initiating treatment, clinical parameters to be monitored, and definitions of treatment failure and non-responders. Experts agreed about acceptable efficacy ranges, stressed the importance of adherence, and recommended follow-up every six months. Simultaneous competitive defocus spectacle lenses were considered the most appropriate optical intervention based on current evidence. Initiation of treatment with simultaneous competitive defocus spectacle lenses was considered appropriate in younger school-age children showing clear evidence of myopia progression, while additional statements provided guidance on the use of low-dose atropine, covering timing and concentration, criteria for discontinuing therapy and subsequent monitoring, and the potential role of combination approaches. On the other hand, new approaches such as red-light therapy were not recommended given the current lack of evidence.

For the epidemiology (5 statements), experts identified the most relevant indicators for monitoring pediatric myopia, including prevalence, annual progression, and age at diagnosis. They recognized the influence of urbanization and school-related behaviours, the increased risk linked to remote schooling during the COVID-19 pandemic, the vulnerability of high-risk subgroups such as children with positive family history or early or intensive exposure to digital devices, and the urgent need for standardized national school-based surveillance protocols.

Regarding etiology and risk factors (5 statements), genetic factors and specific polymorphisms were confirmed as predominant determinants, supported by the clinical relevance of family and clinical history. Accommodation and peripheral hyperopic defocus were considered potential contributors to axial elongation, while outdoor activity and natural light were identified as the most effective preventive strategies, particularly in reducing the onset of myopia rather than its progression.

The original Italian version of the final statements is presented in appendix S1.

### Statements without consensus

Although the Delphi process led to the acceptance of 53 statements, a number of items did not reach the predefined thresholds and were therefore excluded from the final set.

Health policy. No consensus was reached on certain organizational and surveillance strategies, including the operational involvement of primary care pediatricians (62.2%) and screening by orthoptists or ophthalmologists (70.3%). This lack of consensus referred specifically to their operational responsibility in population screening, while the educational and awareness-raising roles of pediatricians and ophthalmologists were consistently supported, as reported in the approved statements.

Economic aspects. Within this domain, no specific item failed to reach consensus, although some heterogeneity in comments was found in how economic evaluations and reimbursement strategies should be implemented at national or regional levels.

Prevention of myopia. Some experts expressed uncertainty about the relative contribution of specific environmental factors (e.g., near work versus digital device use). Although all related items reached more than 65% agreement, none achieved the 80% threshold required for consensus, mainly reflecting variability in how these factors were prioritized rather than disagreement on their overall importance in myopia prevention.

Monitoring and control of myopia progression. Several clinical criteria did not reach consensus: the presence of additional risk factors (55.6%), patient or family compliance (61.1%), maximum age for atropine use (37.8%), and thresholds for discontinuing treatment based on spherical equivalent refraction (54.1%) or axial length (62.2%). The optimal duration of atropine treatment until stabilization achieved 70–73% agreement but did not surpass the threshold. Similarly, efficacy thresholds for ophthalmic lenses based on axial length reduction ≥ 0.2 mm/year did not reach consensus (78.4%). Conditions such as pathological myopia, intensive digital device use, and extended near work were considered potential determinants of lower treatment efficacy, yet the level of agreement (56–76%) remained below the predefined threshold.

Epidemiology. No agreement was obtained on the use of certain indicators, including the prevalence of high myopia (≥ 6D, 45.9%), or on the classification of specific high-risk subgroups such as preterm children (62.2%), children of Asian origin (62.2%), and those with early exposure to electronic devices (75.7%). The panel acknowledged that early or intensive digital device use may increase vulnerability but did not reach consensus on its inclusion as a formal epidemiological indicator of risk. No consensus was achieved on barriers to data collection, including low public awareness (75.7%) and limited involvement of healthcare professionals (56.8%). Proposed predictive variables for risk models, such as school-related habits (67.6%) and screen exposure time (75.7%), also remained below the threshold.

Etiology and risk factors. No consensus was reached on the influence of gender (maximum agreement 65–68%) or on the specific role of several genetic polymorphisms (e.g., GJD2, MYP1, COL2A1, with agreement rates between 20% and 55%). Regarding ocular accommodation, although a role for peripheral hyperopic defocus was recognized (86.5% agreement), other hypotheses such as accommodative lag and visual fatigue did not exceed 60% agreement.

## Discussion

This work represents the first structured national Delphi consensus on pediatric myopia management in Italy, integrating clinical, epidemiological, policy, and economic perspectives. Its contribution lies not in generating new primary evidence, but in translating existing knowledge into context-specific, expert-endorsed recommendations.

The panel reached agreement on 53 statements across six thematic domains, reflecting the specific contextual needs of the Italian healthcare system.

A first important finding concerns health policy and organization of care. Experts emphasized the value of early vision screening at the age of three, in line with existing pediatric recommendations [48–50], and highlighted the need for specialized territorial clinics and multidisciplinary networks [51, 52]. The results highlight the need to better integrate pediatric ophthalmology into local healthcare services, in line with IMI and national society recommendations for early detection and coordinated care.

Regarding economic aspects, the panel emphasized the burden of pathological myopia and its complications, consistent with global estimates of lifetime costs and societal impact [53]. Statements on the cost-effectiveness of low-dose atropine, simultaneous competitive defocus spectacle lenses, and lifestyle interventions are consistent with previous evaluations, while the focus on long-term assessments and the identification of tax deductions as the most equitable measure reflect the Italian reimbursement framework and policy context.

In the domain of prevention, agreement converges on outdoor activity and natural light as key protective factors, while nutrition and supplements are excluded for lack of evidence. The criteria for pre-myopia were consistent with international definitions but applied more cautiously, requiring additional risk factors before starting treatment. This position underlines ethical concerns and the lack of uniform consensus worldwide. For example, the IMI defines pre-myopia as a refractive state of an eye between ≤ + 0.75 D and > − 0.50 D in children when baseline refraction, age, and other quantifiable risk factors suggest a high likelihood of future myopia [31].

Statements on monitoring and control confirmed the role of cycloplegic refraction and axial length as significant parameters and recognized adherence as one of the major determinants of efficacy. An acceptable efficacy range was defined (25–65% reduction vs. controls) and recommended 6-month follow-up intervals.

The axial length threshold reported in the consensus statements reflects the clinical perspective of the panel and should not be interpreted as a uniform efficacy standard applicable to all interventions or follow-up durations.

Simultaneous competitive defocus spectacle lenses, as DIMS spectacle lenses, were endorsed as the most appropriate optical option supported by robust evidence [23], while atropine 0.01% is considered to be the most common starting concentration [54], with treatment usually continued until stabilization. Importantly, experts agreed that no universally recognized thresholds exist for discontinuation, stressing the need for individualized decision-making. Red-light therapy and other emerging technologies were explicitly not recommended, given the limited duration of available studies and the need for robust long-term safety data in pediatric populations before routine clinical adoption [29, 30].

The epidemiology and etiology domains are focus on the importance of standardized school-based surveillance, the role of environmental and behavioral exposures, and the contribution of family history and genetics [55, 56]. Importantly, recent evidence further supports the need to move beyond spherical equivalent refraction alone when assessing risk and progression. In the CLEERE study, axial length trajectories were shown to vary significantly according to age, sex, and ethnicity, reinforcing the concept that biometric parameters provide essential information for risk stratification and early identification of children at higher risk of myopia progression [57].The identification of digital device use as a risk factor, alongside urbanization and reduced time spent outdoors, is consistent with evidence from Asian and European cohorts, including recent data from Italy.

Some statements, however, did not achieve consensus, even after multiple rounds, and are therefore excluded from the final set. These unresolved items mainly concerned the precise thresholds for starting or stopping treatment, the role of demographic subgroups (e.g., prematurity, Asian origin, gender), and additional school or behavioral variables to be included in surveillance or predictive models. Their exclusion is not in contrast with the final statements but marks persistent uncertainties and areas where evidence is still insufficient or heterogeneous.

Areas where consensus was not achieved highlight persistent heterogeneity in clinical practice and identify priority topics requiring further research and methodological clarification.

Agreement on similar clinical priorities, such as as outdoor time, parental education, adherence, and clinical follow-up, are reported in the 2024 UK and Ireland modified Delphi [32], which is focused mainly on practical and multidisciplinary aspects of myopia management. In contrast, the Italian Delphi extended beyond clinical guidance to include economic and policy perspectives, such as national screening programs, specialized territorial clinics, and economic support procedures reflecting the structure and needs of the Italian public health system. Moreover, while the UK/Ireland Delphi involved several professional disciplines (ophthalmologists, optometrists, and orthoptists), this panel consisted exclusively of pediatric ophthalmologists, with a more clinically centered discussion on treatment selection and decision-making.

The more conservative definition of pre-myopia adopted by the Italian panel also reflects a pragmatic and ethically careful orientation, emphasizing individualized assessment over early pharmacological intervention in low-risk children.

The panel emphasized documented progression as an important criterion for treatment initiation within the consensus process. At the same time, clinical decision-making remains individualized and may integrate multiple factors beyond short-term progression alone.

Overall, the Italian Delphi adds a complementary southern European voice to the international effort to harmonize myopia management strategies, highlighting how evidence-based recommendations can be adapted to different healthcare systems and policy frameworks.

### Strengths and limitations

This study has several strengths. First, it achieved a 100% response rate across three Delphi rounds, minimizing the risk of attrition bias. Second, the panel was geographically diverse, ensuring a broad viewpoint on pediatric myopia. Third, the methodology was rigorous, with predefined thresholds, iterative refinement of items, and use of structured feedback.

Consensus represents a strength, but it should be recognised that it is not the highest level in the hierarchy of scientific evidence. Several statements are clearly expert experience and opinion rather than occurred from robust clinical trial data. Indeed, the Delphi method, by design, aggregates expert judgments but cannot compensate for existing gaps in the literature. For instance, the unresolved statements regarding thresholds for initiating or discontinuing therapy, or the inclusion of specific demographic subgroups, remain areas where empirical evidence is not yet sufficiently mature.

Although multidisciplinary, the predominance of pediatric ophthalmologists may have accentuated the clinical perspective while underrepresenting optometric, health policy, economic, and epidemiological viewpoints.

In this context, the absence of a formal consensus statement on orthokeratology and soft contact lenses for myopia control should not be interpreted as a lack of supporting scientific evidence. Rather, it reflects the specific professional composition of the panel, exclusively pediatric ophthalmologists, and the current organization of myopia care in Italy, where contact lens fitting, particularly orthokeratology, is less frequently managed within hospital-based pediatric ophthalmology settings. Consequently, the final statements primarily mirror practices more directly embedded within the Italian public healthcare framework.

The national setting and healthcare structure inevitably influenced the framing and prioritization of statements, potentially limiting their direct transferability to countries with different reimbursement structures or epidemiological profiles.

Response bias or anchoring effects cannot be ruled out: experts may have progressively shifted toward the group median in response to repeated feedback. This tendency, however, is intrinsic to the Delphi process.

Some items approached consensus but were excluded when divergence persisted, a finding that may partly reflect the ceiling effect of predefined thresholds or panel fatigue and convergence pressure. For borderline items, further discussion would be warranted.

Finally, consensus statements should be viewed as a snapshot in time and may require revision as new evidence emerges. Future long-term randomized and observational studies are needed to refine current recommendations. The unresolved items clearly delineate the research priorities that need to be addressed in order to advance future guidance.

This perspective is consistent with international reflections, which emphasize both the urgent need for validated myopia control strategies and the persistence of unresolved questions requiring further research [58].

## Conclusion

These consensus statements can inform not only clinical practice but also guide health policy planning in Italy. For clinicians, they provide recommendations on screening, risk stratification, treatment initiation, monitoring, and therapeutic choices. For policymakers, they highlight the need for school-based surveillance, financial support, and national strategies.

More generally, the findings contribute to the international dialogue on harmonizing approaches to pediatric myopia, while also identifying specific research priorities such as optimal atropine dosing, the long-term effectiveness of combination therapies, and strategies to improve adherence.

Equally important are the areas where consensus was not achieved, which emphasize the complexity of pediatric myopia and the need for further investigation, particularly regarding treatment thresholds, subgroup risk factors, and surveillance strategies.

Coordinated, multicenter research efforts, including randomized clinical trials, observational studies, and updated Delphi studies in the next years, will be needed to strengthen the evidence and support the development of robust clinical guidelines and public health policies.

## Supplementary Information

Below is the link to the electronic supplementary material.

Supplementary Material 1

Supplementary Material 2

## Data Availability

No datasets were generated or analysed during the current study.
