# Supplementary material for: Italian consensus on pediatric myopia: findings from a three-round modified delphi study
Source: Graefes Arch Clin Exp Ophthalmol. 2026 Apr 30;264(10):3215–29. doi: 10.1007/s00417-026-07246-9 (PMC13635050; doi:10.1007/s00417-026-07246-9)
Supplement: Supplementary file 2 [file 417_2026_7246_MOESM2_ESM.docx]

**Italian consensus on pediatric myopia: Findings from a three-round modified Delphi study**

***(Schiavetti I et al., 2025)***

**Final statements**

**POLITICHE SANITARIE**

1. Introdurre lo screening visivo all'età di tre anni è utile per la possibilità di diagnosticare e trattare precocemente deficit visivi come miopia, ambliopia e strabismo, e per identificare precocemente fattori di rischio come familiarità o uso scorretto dei dispositivi digitali.
2. L'accesso alle cure oculistiche per i bambini con miopia può essere migliorato attraverso l'implementazione di ambulatori specialistici territoriali e la creazione di reti di specialisti multidisciplinari in ambito pediatrico.
3. Le campagne di sensibilizzazione nazionali sono utili per ridurre la progressione della miopia e dovrebbero mirare a informare famiglie, scuole e pediatri sui rischi legati alla miopia e sulle strategie di prevenzione, promuovendo anche stili di vita salutari come l'aumento del tempo trascorso all'aperto, la limitazione del lavoro da vicino e l'uso appropriato dei dispositivi digitali. Tali campagne dovrebbero inoltre includere informazioni sui fattori di rischio e sulle possibili complicanze associate alla miopia, al fine di migliorare la consapevolezza e il coinvolgimento della popolazione.
4. Oltre alle detrazioni fiscali per occhiali e lenti a contatto, dovrebbero essere previsti finanziamenti pubblici a sostegno di programmi finalizzati alla prevenzione e alla gestione della miopia.
5. Oculisti e pediatri di libera scelta dovrebbero essere riconosciuti come promotori chiave di campagne scolastiche per sensibilizzare sulla miopia e incentivare abitudini visive sane nei bambini.
6. La presenza di miopia in bambini e adolescenti può incidere significativamente sull'autostima, sulle relazioni sociali e sulle attività quotidiane o scolastiche.
7. I pediatri di libera scelta dovrebbero avere un ruolo centrale nell'educazione dei genitori alla salute visiva dei bambini.
8. Una dichiarazione congiunta da parte delle società scientifiche è uno strumento utile per aumentare la consapevolezza dei pediatri sull'importanza dello screening visivo precoce.

**ASPETTI ECONOMICI**

1. Le visite oculistiche regolari, i dispositivi correttivi (come occhiali e lenti a contatto) e i trattamenti farmacologici e ottici (come l'atropina a basso dosaggio, l'ortocheratologia e le lenti a defocus competitivo simultaneo) sono tra i principali costi diretti associati alla miopia.
2. I costi legati alle visite oculistiche regolari e ai trattamenti per la riduzione della progressione miopica sono un investimento preventivo giustificato.
3. Il burden economico della miopia patologica ha un impatto rilevante sia sulla qualità della vita sia sui costi sanitari, e dovrebbe essere una priorità nelle strategie di prevenzione e gestione.
4. I costi indiretti della miopia, come l'impatto sulla carriera o l’assistenza informale, non sono adeguatamente considerati nelle attuali valutazioni economiche.
5. Le complicanze della miopia elevata, come la degenerazione maculare miopica, il distacco di retina, la neovascolarizzazione coroideale miopica (CNV) e il danno glaucomatoso, hanno il maggiore impatto sulla qualità della vita dei pazienti e generano i più elevati costi diretti e indiretti per il sistema sanitario.
6. Le lenti a defocus competitivo simultaneo sono tra le strategie più costo-efficaci per la gestione della miopia nei bambini e rappresentano una delle innovazioni tecnologiche più promettenti per ridurne l'impatto economico. Anche l'atropina a basso dosaggio e le modifiche dello stile di vita, come l'aumento del tempo trascorso all'aperto e la riduzione delle attività da vicino, sono interventi costo-efficaci.
7. Valutazioni economiche a lungo termine, che tengano conto della prevenzione delle complicanze e dei risparmi futuri, sono fondamentali per stimare la reale costo-efficacia dei trattamenti emergenti per la miopia.
8. Le detrazioni fiscali per spese sanitarie (incluse visite oculistiche, correzioni ottiche e trattamenti farmacologici) sono la misura economica più efficace per migliorare l'accesso sia ai trattamenti convenzionali che a quelli innovativi per la miopia.

**PREVENZIONE DELLA MIOPIA**

1. Raccomandare da 1 a 2 ore al giorno di attività all'aperto è una misura preventiva appropriata per ridurre l'insorgenza della miopia nei bambini.
2. L'esposizione alla luce naturale contribuisce alla prevenzione della miopia stimolando il rilascio di dopamina retinica, che svolge un ruolo protettivo contro l'allungamento assiale.
3. Limitare il tempo ricreativo trascorso davanti agli schermi, promuovere comportamenti visivi corretti (come una postura adeguata, una distanza di lettura appropriata ed evitare l'uso scorretto dei dispositivi digitali) e aumentare il tempo trascorso all'aperto sono strategie fondamentali per ridurre i rischi associati al lavoro da vicino nei bambini. Tali misure dovrebbero essere sostenute anche in ambito scolastico, attraverso programmi educativi, modifiche dell'organizzazione degli spazi e delle attività, e la riduzione dell'uso non essenziale degli schermi durante l'orario scolastico.
4. In ambito scolastico, sensibilizzare studenti, insegnanti e famiglie sull'importanza di corrette abitudini visive è essenziale per una prevenzione efficace della miopia.
5. In accordo alla definizione IMI, un valore refrattivo compreso tra +0.75 D e −0.50 D, o inferiore alla media per l'età, può rappresentare pre-miopia. Tuttavia, l'avvio di un trattamento è eticamente appropriato solo in presenza di fattori di rischio individuali ben definiti, come parametri biometrici alterati o una forte familiarità.
6. Il rispetto dei ritmi circadiani e un adeguato riposo notturno sono fattori utili nella prevenzione della miopia.
7. Attualmente non vi sono evidenze scientifiche solide a supporto di un ruolo della nutrizione o degli integratori alimentari nella prevenzione della miopia.

**MONITORAGGIO E CONTROLLO DELLA PROGRESSIONE MIOPICA**

1. La decisione di iniziare un trattamento per la progressione miopica dovrebbe basarsi su una combinazione di fattori, tra cui un tasso di progressione significativo, l'età del paziente, la familiarità per miopia, il tipo di miopia (es. elevata o patologica), la presenza di ulteriori fattori di rischio, la disponibilità e l'aderenza del paziente e della famiglia, nonché l'esistenza di linee guida o raccomandazioni cliniche specifiche.
2. I principali parametri clinici da monitorare per valutare l'efficacia del trattamento sono la refrazione sferica equivalente in cicloplegia e la lunghezza assiale.
3. Un tasso di progressione della lunghezza assiale superiore ai valori attesi nonostante il trattamento è un forte indicatore di fallimento terapeutico e rappresenta il criterio più rilevante per identificare un paziente come non responder alla terapia per il controllo della miopia.
4. Un trattamento è inefficace quando la progressione annua in refrazione sferica equivalente supera i valori target e/o quando la lunghezza assiale aumenta oltre le soglie attese.
5. I pazienti in trattamento dovrebbero essere sottoposti a controlli circa ogni 6 mesi.
6. Secondo la letteratura, i range di efficacia accettabili per i trattamenti sono: riduzione minima della progressione miopica del 25–35%, massima del 55–65%, rispetto ai controlli non trattati.
7. Uno dei principali fattori che riduce l'efficacia dei trattamenti (ottici o farmacologici) è la scarsa aderenza.
8. La scelta del tipo di lenti oftalmiche per il controllo della miopia dovrebbe basarsi principalmente su evidenze scientifiche pubblicate e raccomandazioni delle società scientifiche.
9. Le lenti oftalmiche a defocus competitivo simultaneo rappresentano la tecnologia ottica più appropriata per il controllo della miopia nei bambini, in quanto attualmente sono l'unico tipo di lente supportato da evidenze scientifiche solide.
10. L'inizio del trattamento con lenti oftalmiche a defocus competitivo simultaneo è appropriato nei bambini a partire dai 6–8 anni di età con progressione miopica ≥0.50 D/anno (valutata su un periodo di 6–12 mesi) e/o aumento della lunghezza assiale ≥0.2 mm/anno.
11. Non esiste una soglia fissa in refrazione sferica equivalente o lunghezza assiale universalmente riconosciuta per l'interruzione del trattamento con lenti a defocus competitivo simultaneo.
12. Una riduzione assoluta del tasso annuo di progressione della miopia di almeno 0,50 D in refrazione sferica equivalente e/o di almeno 0,2 mm all'anno nella lunghezza assiale rappresenta la soglia per definire una lente come efficace nel controllo della miopia.
13. È accettabile sospendere l'uso degli occhiali per il controllo della miopia durante le attività sportive per un massimo di due ore, fino a tre volte a settimana.
14. L'atropina allo 0,01% è la concentrazione iniziale più comunemente utilizzata per il controllo della miopia, sebbene non esista una strategia standardizzata per l'aggiustamento del dosaggio nel tempo e la pratica clinica sia eterogenea. La scelta della concentrazione è guidata principalmente dalle evidenze scientifiche disponibili e il trattamento viene generalmente proseguito fino alla stabilizzazione della miopia.
15. Non esiste un’età massima universalmente accettata per l’inizio del trattamento con atropina a basso dosaggio, mentre l’inizio può essere considerato già a partire dai 4–5 anni, ma in pratica è più frequentemente avviato tra i 6–8 anni in presenza di una progressione documentata della lunghezza assiale ≥0.2 mm/anno. Tuttavia, la decisione dovrebbe basarsi su una valutazione clinica personalizzata, che includa la velocità di progressione, l’età e la storia familiare, piuttosto che su soglie fisse di refrazione sferica equivalente. L’uso esclusivo dell’atropina in caso di progressione ≥0.5 D/anno rimane controverso.
16. L'etnia e la storia familiare possono influenzare la decisione in merito alle soglie di età per iniziare o terminare il trattamento con atropina.
17. Dopo la sospensione del trattamento con lenti a defocus competitivo simultaneo o atropina, è raccomandabile programmare controlli ogni sei mesi per monitorare l'eventuale progressione della miopia.
18. Oltre ai parametri di progressione, l'età del paziente e la storia familiare di miopia sono fattori clinici importanti che guidano la scelta tra atropina e lenti a defocus competitivo simultaneo.
19. La terapia combinata (ad esempio, atropina e lenti a defocus competitivo simultaneo) è appropriata in caso di risposta inadeguata alla monoterapia e dovrebbe essere personalizzata in base alle caratteristiche cliniche, come la progressione rapida o la miopia di partenza elevata.
20. La terapia con luce rossa e altri trattamenti emergenti non sono attualmente raccomandati nella pratica clinica, a causa delle evidenze ancora insufficienti.

**EPIDEMIOLOGIA**

1. La percentuale di bambini con diagnosi di miopia, la proporzione di quelli che presentano una progressione superiore a 1 diottria all'anno e l'età media alla diagnosi sono tutti indicatori epidemiologici rilevanti per monitorare la prevalenza e l'andamento della miopia pediatrica.
2. L'andamento della miopia in età pediatrica è fortemente influenzato da fattori ambientali e comportamentali associati all'urbanizzazione, così come da abitudini scolastiche quali la riduzione del tempo all'aperto e il prolungato lavoro da vicino.
3. I cambiamenti legati alla didattica a distanza, come avvenuto durante la pandemia da COVID-19, hanno significativamente aumentato il rischio di insorgenza e progressione della miopia. Negli studi longitudinali, è fondamentale raccogliere metriche come la refrazione in cicloplegia, la lunghezza assiale, il tempo trascorso all'aperto e la frequenza e durata dell'uso di dispositivi digitali in ambito scolastico. Inoltre, per sviluppare modelli predittivi affidabili, è importante includere dati su esposizione agli schermi, età alla diagnosi, progressione annua della miopia, fattori ambientali e familiarità o predisposizione genetica.
4. I bambini con una storia familiare positiva per miopia e quelli esposti precocemente o intensivamente ai dispositivi digitali sono i sottogruppi più vulnerabili allo sviluppo della miopia.
5. La sorveglianza epidemiologica è una strategia a supporto della prevenzione della miopia patologica. In particolare, i programmi di screening scolastico sono il contesto più efficace per implementare l'identificazione precoce e la raccolta standardizzata dei dati. Tuttavia, l'assenza di protocolli nazionali standardizzati rappresenta ancora un ostacolo rilevante alla raccolta sistematica di dati sulla miopia in Italia.

**EZIOLOGIA E FATTORI DI RISCHIO**

1. L'insorgenza della miopia è influenzata da fattori genetici, dall'esposizione alla luce naturale, dal tempo trascorso all'aperto e dal lavoro da vicino.
2. Polimorfismi genetici specifici localizzati in determinati loci genomici sono associati all'insorgenza della miopia.
3. Una storia familiare e/o clinica sistemica dettagliata è una valutazione clinica utile per decidere se inviare un bambino con miopia ad approfondimenti specialistici.
4. L'accomodazione oculare può contribuire al rischio di sviluppo della miopia. Un ulteriore meccanismo sembra coinvolgere il defocus ipermetropico periferico, che può stimolare l'allungamento assiale. Quando l'accomodazione ha un ruolo, le strategie preventive mirate alla sua gestione sono considerate rilevanti.
5. Trascorrere tempo all'aperto ed esporsi alla luce naturale sono strategie riconosciute per prevenire l'insorgenza della miopia. Il tempo all'aperto risulta particolarmente efficace nel prevenire l'esordio della miopia, più che nel rallentarne la progressione.
